# Synergizing Safety: A Customized Approach to Curtailing Unplanned Extubations through Shared Decision-making in the NICU

**DOI:** 10.1097/pq9.0000000000000729

**Published:** 2024-05-09

**Authors:** Parvathy Krishnan, Nilima Jawale, Adam Sodikoff, Susan R. Malfa, Kathleen McCarthy, Lisa M. Strickrodt, Diana D’Agrosa, Alexandra Pickard, Lance A. Parton, Meenakshi Singh

**Affiliations:** * From the Department of Neonatal-Perinatal Medicine, Westchester Medical Center, and New York Medical College, Valhalla, N.Y.; † Department of Pediatrics, State University of New York Upstate Medical University.

## Abstract

**Background:**

Unplanned Extubation (UE) remains an important patient safety issue in the Neonatal Intensive Care Unit. Our SMART AIM was to decrease the rate of UE by 10% from the baseline from January to December 2022 by emphasizing collaboration among healthcare professionals and through the use of shared decision-making.

**Methods:**

We established an interdisciplinary Quality Improvement team composed of nurses, respiratory therapists, and physicians (MDs). The definition of UE was standardized. UE was audited using an apparent cause analysis form to discern associated causes and pinpoint areas for improvement. Interventions were implemented in a step-by-step fashion and reviewed monthly using the model for improvement. A shared decision-making approach fostered collaborative problem-solving.

**Results:**

Our baseline UE rate was 2.3 per 100 ventilator days. Retaping, general bedside care, and position change accounted for over 50% of the UE events in 2022. The rate of UE was reduced by 48% by the end of December 2022. We achieved special-cause variation by the end of March 2023.

**Conclusions:**

The sole education of medical and nursing providers about various approaches to decreasing unnecessary retaping was ineffective in reducing UE rates. Shared decision-making incorporating inputs from nurses, respiratory therapists, and MDs led to a substantial reduction in the UE rate and underscores the potential of systematic evaluation of risk factors combined with collaborative best practices.

## INTRODUCTION

Unplanned extubation (UE) in the Neonatal Intensive Care Unit (NICU) remains a persistent and significant safety hazard. Such events rank as the fourth most reported adverse occurrences in United States NICUs.^1^ UEs are typically described as unplanned, or unexpected extubations that are not clinically indicated or ordered by the clinical provider.^2^ The complexity and vulnerability of the NICU population make them susceptible to various factors that enhance UE risk. Among these are prolonged mechanical ventilation, limited surface for securing endotracheal tubes (ETT), challenges with secretions and emesis, patient size, the fragile skin of premature infants, the use of uncuffed ETTs, and inconsistent sedation practices for intubated neonates.^3–6^

The repercussions of UE are immediate and significant. Rapid reintubation post UE exposes neonates to a higher likelihood of associated complications compared with elective or nonemergency scenarios.^7^ Short-term complications of UE include acute cardiorespiratory events, complications related to bag valve and mask ventilation, and risks of injury related to repeated intubations.^3,8^ Beyond these, UEs can culminate in severe outcomes such as hemodynamic destabilization, airway trauma, intraventricular hemorrhages, extended hospital stays, prolonged mechanical ventilation, acquired subglottic stenosis, and even mortality.^9–12^

Quality improvement (QI) endeavors have shed light on the effectiveness of diverse strategies in curtailing UE rates.^13^ Some of these approaches have focused on bundles addressing standardization of ETT position and securement, root cause analysis, establishing protocols for high-risk situations in intubated patients, patient positioning, and the use of lean methodology.^9,10,14–16^ However, enforcing a systematic, all-encompassing approach in busy NICUs, particularly those bereft of prior audit systems, poses significant challenges.^17,18^ Central to our study’s novelty is the integration of a shared decision-making approach, guiding our efforts to address challenges and foster collaborative problem-solving.^19^ Applying the concept of shared decision-making to UE rate reduction, we delineate our journey in integrating best practice components and introducing interventions, while avoiding staff overextension or routine sedation/restraint employment. Our SMART aim was to decrease the rate of UE by 10% from the baseline within 12 months, spanning from January to December 2022.

## METHODS

### Context

The NICU at Maria Fareri Children’s Hospital is a 54-bed level-IV NICU that provides care for infants with complex medical needs, including those requiring short-term and prolonged intubation due to conditions such as extreme prematurity, pulmonary hypertension, hypoxic-ischemic encephalopathy, and congenital anomalies. Before the initiation of this QI project in quarter one 2021, there was no formal system to audit or minimize the UE occurrences.

### Team

Establishing a team to implement the QI initiative was a crucial step in our project. The first step was to identify key stakeholders who would drive the initiative forward. The stakeholders were selected from different strata to ensure representation from various departments and expertise. The identified champions included respiratory therapists (RT), nurses, fellows, nurse practitioners, and an attending physician.

### Interventions

The team focused on designing and implementing a formal system to prevent UE. The definition of UE was standardized. An UE was defined as unintended dislodgement of the ETT from the trachea in an infant receiving mechanical ventilation.^20^ The team developed and implemented interventions to mitigate UE, and regular audits were performed to identify potential areas for improvement.

Our first step was to identify all instances of UE using a data collection tool, the apparent cause analysis (ACA) form. Continuous monitoring of UE events allowed the team to promptly detect and respond to UE incidents. Rather than filling out the form retrospectively, as had been done in the past, we implemented a practice of completing the ACA form in real-time with the nurse, RT, and medical provider as a team after each UE. The signature of all three providers on the form was a surrogate marker of adherence to this intervention. This practice allowed the team to determine the causes of each UE. Data collected included the event date, reintubation details, history of prior UE, type of intubation (nasal versus oral), type of mechanical ventilation (conventional versus high frequency), ETT securement details, taped location of the ETT (corner, the center of the mouth, nasal), presence of suction apparatus, phase of treatment (acute, weaning or awaiting extubation), sedation, staff ratio, contributors, and UE outcome. The completed ACA form was used to identify the leading causes of UE (Fig. 2).

**Fig. 1.**
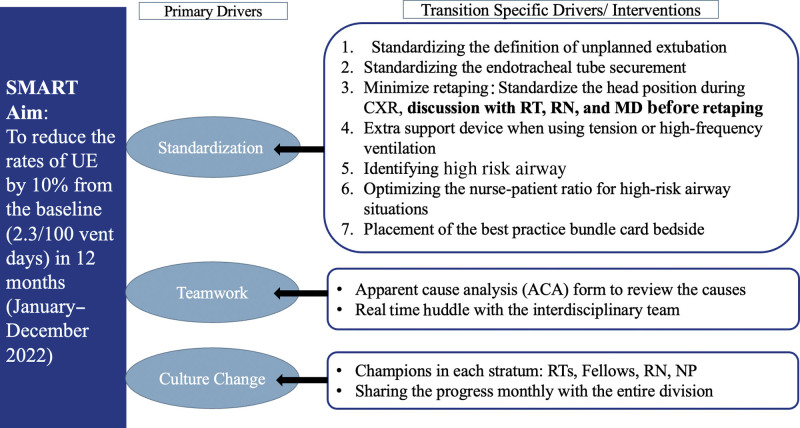
Key driver diagram. RN: registered nurse, MD: doctor of medicine, NP: nurse practitioner.

We established monthly meetings with the QI team to review the ACA UE forms and identify key areas for improvement. Our initiative used a specific bundle designed for our context, which was selected based on the most prevalent causes for UE identified by the ACA form. A key driver diagram was created to outline our approach (Fig. 1). The plan-do-study-act model for improvement was used to implement the process changes based on the most prevalent causes of UE.^21^ Interventions were introduced in a step-by-step fashion and reviewed monthly.

### Measures

We used the overall rate of UE as the primary outcome measure, defined as the number of UE events per 100 ventilator days. To ensure the consistency of our data collection, we obtained information on the total number of ventilator days per month through daily audits. We included all patients with an ETT but excluded those with tracheostomy tubes. Compliance with the interventions were measured through monthly audits conducted by the nursing. (**Supplemental figure 1,** http://links.lww.com/PQ9/A555.)

### Analysis

We used Microsoft Excel and QI Macros for data analysis. The causes of UEs were periodically analyzed using Pareto charts. To examine the monthly rate and detect special-cause variation, we created run and control charts in accordance with the American Society for Quality special-cause rules.^22^ The institutional review board at the New York Medical College granted an exemption for this QI initiative because it involved the implementation and evaluation of practices aimed at improving the quality of patient care.

## RESULTS

### Baseline Data

Our baseline UE rate was calculated from March 2021 to March 2022. The UE per month ranged from 0 to 14 events. The number of ventilator days/ month ranged from 61 to 437 days. Our baseline UE rate was 2.3 per 100 ventilator days. (**Supplemental figure 2,** http://links.lww.com/PQ9/A556.)

**Fig. 2.**
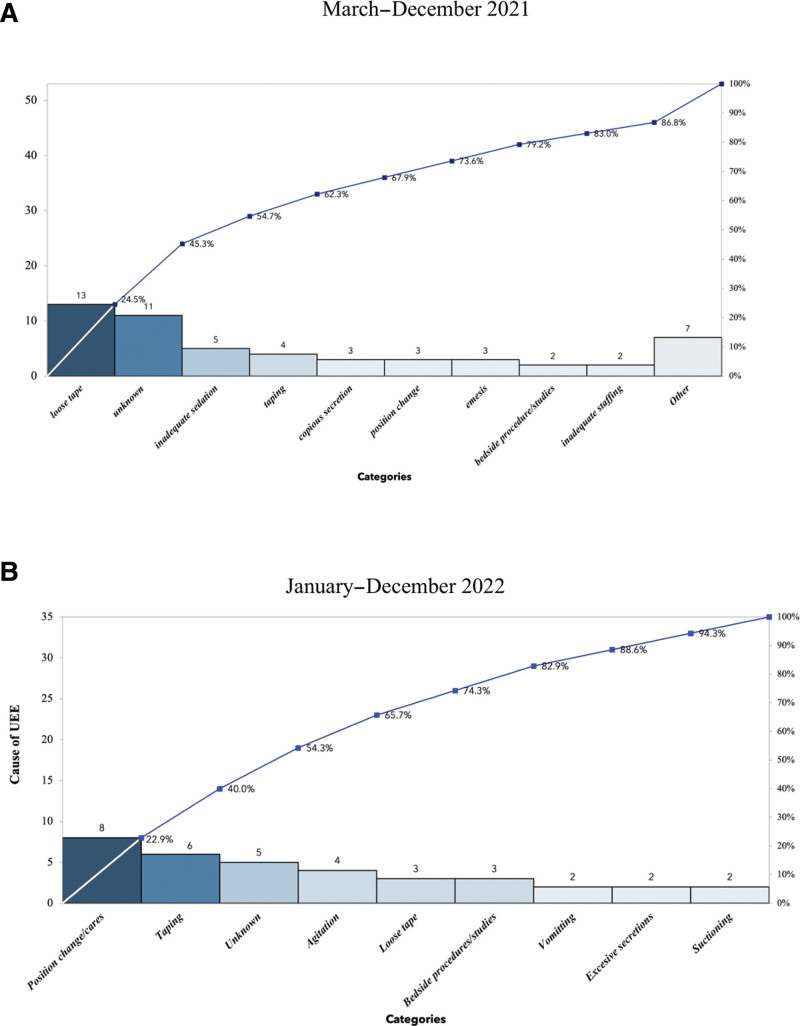
Pareto chart: displaying reasons for unplanned extubation (UEs). The number of UE is on the left vertical axis. The right vertical axis shows the cumulative percentage for each reason noted for UE. A, March–December 2021: loose tape, unknown causes, and inadequate sedation represented 54% of the reasons noted for UE. B, January–December 2022: position change/care, taping, and unknown factors represented 54% of the reasons noted for UE.

Loose tape, agitation, and retaping were identified as the primary contributors to more than 50% of the UE events. Other potential contributing factors included increased secretions, patient position changes, suctioning, and agitation (Fig. 2). Reintubation within 1 hour was required in 86% of UE events.

### Outcomes

Interventions were implemented in a phased manner starting in March 2021. Analysis of the ACA forms obtained in 2021 revealed that over 24% of the UE events were attributed to loose tape (Fig. 2). As a result, our initial interventions aimed to reduce the incidence of loose tape by standardizing the ETT securement. The process of securing the ETT involves the unit RT and nurse undertaking this task together after the medical provider has intubated the infant. Our observation indicated significant variation in tube securement practices. Our intervention was designed to standardize the holding and securement of the ETT. The standardized technique involved holding the ETT securely against the hard palate of the infant’s mouth using a finger by one provider, while the other provider secured the tube with tape in a spiral “candy-cane” fashion, extending at least 3 cm from the insertion depth. This method aimed to increase the adhesion surface area and prevent the ETT from loosening or dislodging from the tape. This intervention initially resulted in a decrease in the incidence of loose tape as a cause of UE, as well as an overall reduction in UE rates for the following 4 months. However, in subsequent months, UE rates increased, despite a reduction in the occurrence of loose tape. ACA forms identified retaping mainly done to reposition the ET tube to optimal mid-tracheal depth and dislodgements associated with bedside procedures became the new leading causes of UE in 2022 (Fig. 2).

In 2022, we conducted further interventions to reduce UE occurrences caused by retaping despite nursing and medical provider education. Our investigation revealed that frequent retaping was higher among extremely low birth weight infants due to variable ETT positioning identified during chest X-ray (CXR). This was caused by differing head positions when CXRs were being taken. For example, head flexion or extension or variations with the gel pillow neck support position caused the tube to slide up or down the trachea. These variations resulted in different ETT positions, necessitating repeated retaping. To address this issue, we implemented a standardized approach to ensure midline head positioning during CXR. We also encouraged using an extra support device, called an “angel arm,” to securely and consistently hold the ETT in position, especially for cases requiring tension. (**Supplemental figure 3,** http://links.lww.com/PQ9/A557.)

Despite these process changes and repeated reeducation of providers to consider the appropriate head position while determining the ET position, retaping occurrences were frequent and associated with UE occurrences. It became evident that medical providers were frequently at the helm of retaping decisions. To foster more balanced decision-making, we introduced a retaping huddle before any ETT retaping decision was codified. This huddle, acting as a shared decision-making model, brought the RT, nurse, and medical provider together. The triad then deliberated on the ETT’s position as observed on CXR, the infant’s head positioning, the tension exerted, and the infant’s overall clinical condition. Based upon this huddle, a decision was made regarding whether to reposition the ETT.

**Fig. 3.**
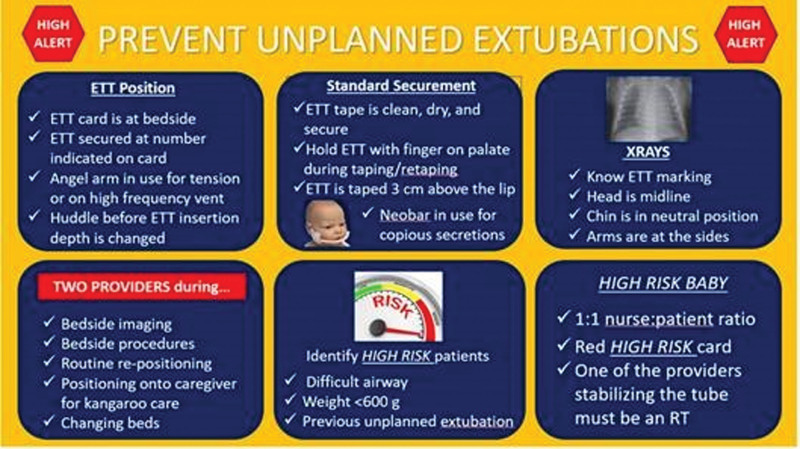
Alert card with components of the best practice bundle.

Our data analysis from the latter quarter of 2022 highlighted another pattern: UEs predominantly transpired around bedside care and procedures. To mitigate this, we instituted guidelines demanding the presence of at least two providers during critical bedside activities, such as imaging, procedures, infant repositioning, kangaroo care transitions, or bed changes. For high-risk patients such as infants with challenging airways due to anatomical variations, infants with a weight under 600 g, or those with prior UE instances, we recommended a 1:1 nurse-patient ratio to enhance supervision and care. However, maintaining a 1:1 ratio was contingent on staffing availability and the needs of the unit. Ultimately, alert cards were implemented at the bedside of every intubated patient with information such as the size and the depth of the ETT, standard ETT securement practices, and instruction for the appropriate CXR head position (Fig. 3). The alert cards were color-coded for easy recognition, with red cards indicating high-risk patients and yellow cards for other intubated patients.

**Fig. 4.**
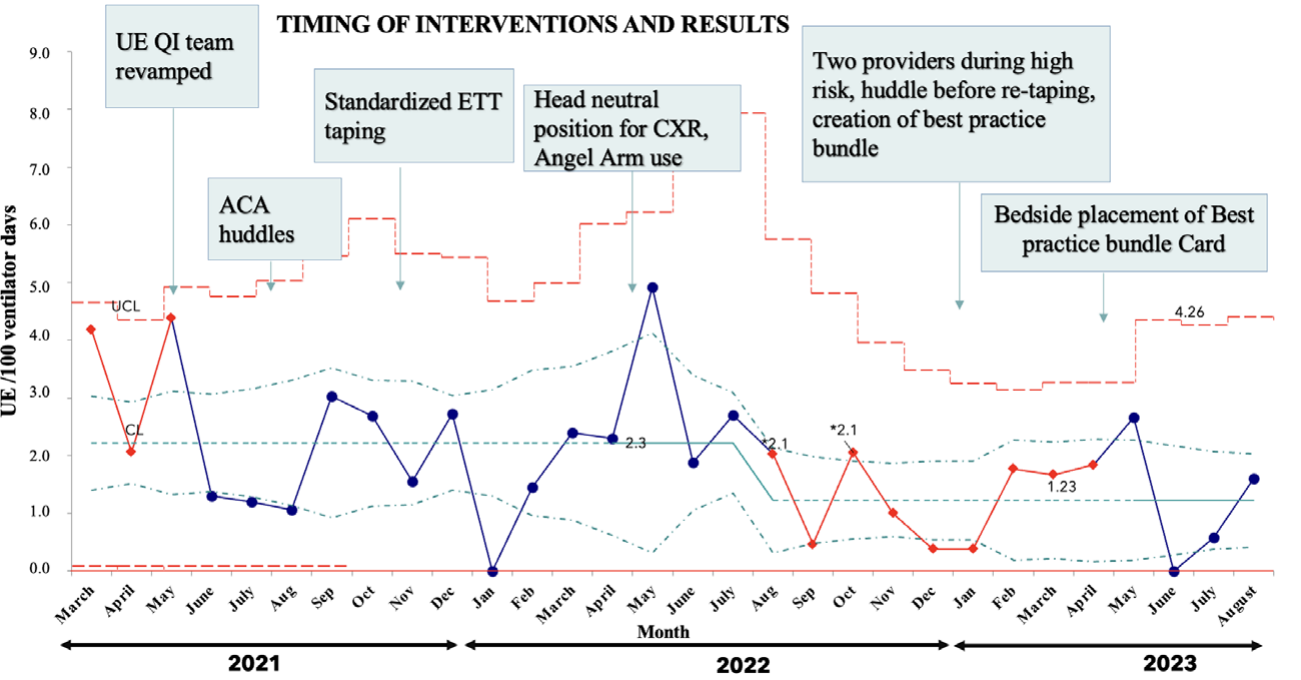
Results: statistical process control chart (u chart) depicting timing of interventions and rate of unplanned extubation (number of unplanned extubation events per 100 ventilator days) from March 2021 to August 2023. There was an aggregate 49% reduction in UE events, from a baseline rate of 2.3 UEs per 100 ventilator days to 1.2 UEs per 100 ventilator days. The special-cause variation is depicted by the red solid line. Shift was determined by statistical process control chart rule of 8 or more points below the baseline. The shift began in August 2022.

By the end of 2022, we observed a significant reduction in the rate of UE. From August 2022, the rates remained below 2.3 per 100 ventilator days for over 8 consecutive months, indicating special cause variation and a centerline shift of the mean UE rate^23^ (Fig. 4). The UE rate settled at 1.2 per 100 ventilator days. There was a confluence of interventions culminating with a significant reduction in UE attributed to retaping interventions and implementation of the retaping huddles. Reduction in retaping occurrences directly mirrored the decrease in UE incidents. The aggregate rate of UE was reduced by 48% by the end of December 2022, from 2.3 in 2021 to a sustained rate of 1.2 per 100 ventilator days.

## DISCUSSION

The successful implementation of a QI initiative in a medical setting requires not just the introduction of procedural changes but also a paradigm shift in how staff view, interact with, and challenge systemic vulnerabilities. Our findings highlight the power of a sequential, multidisciplinary strategy in realigning these vulnerabilities.^(24,25)^ This approach helped us achieve a significant reduction in the UE rate.

The prevalence of UE in NICUs exhibits considerable variability, oscillating between <1 and >5 instances per 100 ventilator days. While the Vermont Oxford Network in 2012 proposed an upper limit of under 2 UE events per 100 ventilator days, other entities argue for even stricter thresholds, at <1 event per 100 ventilator days.^10,14,26^ Many NICUs have adapted best practice bundles to achieve this goal.^7,10,27–29^ In a large multicenter study, Klugman et al described a 17.6% reduction in UE rates in the NICU (1.555 UEs per 100 ventilator days to 1.282 UEs per 100 ventilator days) in their multicenter study, after implementing a bundle. The unplanned extubation rates were higher in the NICU (ranging from 0.6 to 4.5 UE per 100 ventilator days) than in other ICUs.^10^ However, the bundle was implemented at once instead of sequentially, which can tax the caregivers and may influence compliance.^17,18^

At the core of our approach lies the shared decision-making, which is fortified by the meticulous completion of the ACA form by the entire clinical team. It is imperative to recognize that individual perceptions regarding UE may vary within the team. Thus, fostering open discussions and diligently completing the ACA form enables us to swiftly pinpoint any gaps in adhering to current process measures and promptly identify the actual cause of UE.

Our study found that loose tape and retaping of the ETT were the most common causes associated with UE in our population, consistent with earlier studies.^11,16^ Although SPS data underscore the importance of standardized anatomic reference points and securement methods for taping the ETT,^10^ there are currently no standardized guidelines for holding or securing the ETT, and securement practices vary across NICUs. Although some units use ETT securing devices such as neo bars, their use alone does reduce the rate of UE.^9,30^ Fontanez Nieves et al reported a reduction of over 50% in their UE rates by introducing a standardized taping method. However, their study had a much lower number of total ventilator days, and the baseline rate was higher than typically described in the literature (16.1–4.5 per 100 ventilator days).^15^ We recognize that securement practices vary across NICUs, but our intervention involved introducing a standardized taping method. Unlike some previous studies, we implemented this change in a unit with a relatively high baseline rate, demonstrating its effectiveness even in challenging conditions. Our unit uses 3M multipore tape to secure the ETT, but the securement method varied among different staff. To address this issue, we standardized the ETT securement method with better stabilization of the tube.

Some studies show that UE rates are decreased by having an optimal nurse-patient ratio.^5,31,32^ Although maintaining a 1:1 ratio for all intubated patients can be demanding in busy units, the key lies in identifying higher-risk infants who require this level of attention without overburdening staffing resources. By strategically triaging high-risk patients, 1:1 ratio can be effectively allocated to those who need it the most, promoting enhanced patient safety without undue strain on the staff.

The novel aspect of our work is the sequential implementation and the shared decision-making approach. ETT tip position in CXR is influenced by various factors, including head position, ETT tension, and the angle of the CXR. We recognize that differing perceptions among providers regarding the optimal ETT position often leads to frequent retaping and, subsequently, UE. To address this issue, we proposed a model of shared decision-making, uniting the expertise of RTs, nurses, and medical providers in meaningful discussions about ETT positioning in relation to hea placement, tension application, and the infant’s clinical status. This practice cultivated a safety-driven culture within the NICU, instilling a sense of ownership and empowerment in nursing and RT staff. Traditionally, in many NICUs, the authority rests heavily with medical providers, sometimes at the expense of frontline caregivers feeling overshadowed. Our interventions were designed to promote a cultural change emphasizing the importance of shared decision-making, thus empowering nurses and RTs. Our experience shows that this decentralization and shared decision-making model rather than single ownership can develop a culture of constant vigilance and effectively reduce our retaping occurrences and the rate of UE.

To sustain this improvement, we emphasize the necessity of instilling a culture change. We advocate for the development and continuous monitoring of systems based on the tenets of standardization and shared decision-making. By embracing this approach, we foster a lasting culture of patient safety and collective excellence within our unit, ultimately improving the overall quality of care for the infants in the NICU.

### Limitations

Despite the significant reduction in UE rates, our QI initiative was limited. Firstly, it is essential to contextualize our study as a single large regional perinatal center with full-time RTs and ample nursing staff. Consequently, the interventions we successfully implemented may pose challenges in smaller NICUs with more limited resources.

Secondly, although we introduced our customized bundle in a sequential manner, we did not collect data on compliance with each component due to resource limitations. Hence, the lack of compliance data for individual components introduced in sequence restricts limits our capacity to attribute the reduction in unplanned extubation rates solely to the sequentially implemented interventions. It is plausible that a higher compliance rate with specific intervention might have resulted in a substantial reduction in UE rates. We acknowledge the importance of compliance data and recognize its impact on the interpretation of our results.

Additionally, we acknowledge the multifaceted nature of UE, and our focused efforts aimed to minimize the most common contributing factors within our unit. However, we are also mindful that other factors might now assume significance in UE occurrence. Studies have demonstrated the duration of endotracheal intubation as the sole independent risk factor of UE,^4,15^ underscoring the need for timely extubation. To address this, we anticipate the implementation of an extubation readiness protocol, which may optimize the timing of extubation in our high-risk population.^33^ Additionally, we have not yet explored the use of sedation in our NICU population. Because agitation can contribute to UE, careful consideration of analgesia/sedation in high-risk patients is warranted. Finally, we did not include staff surveys or specific quantitative measurements of teamwork. We believe that the culture change we aimed to foster, centered around shared decision-making and a safety-driven mindset, is qualitatively reflected in the reduced retaping occurrences, compliance to the ACA form details, communication, and staff empowerment, demonstrating our approach’s positive impact. Future research may include more formal quantitative assessments to provide a more comprehensive evaluation of these aspects.

Despite the limitations, our project has achieved a significant reduction in the rate of UE. Therefore, we are dedicated to further plan-do-study-act cycles to introduce an extubation readiness protocol and sedation guidelines for our high-risk patients, aiming to achieve even greater improvements in the rate of UE.

## CONCLUSIONS

This QI initiative provides a roadmap to tackle a serious safety event in the NICU with a well-organized and phased QI tool. It underlines the importance of shared decision-making to tackle long-standing system problems and improve outcomes.

## Supplementary Material



## References

[1] Children’s Hospitals’ Solutions for Patient Safety. Unplanned extubations. Available at https://www.solutionsforpatientsafety.org/unplannedextubations. Accessed October 18, 2023.

[2] Mahaseth M Woldt E Zajac ME. Reducing unplanned extubations in a level IV neonatal intensive care unit: The elusive benchmark. Pediatr Qual Saf. 2020;5:e337. 33575517 10.1097/pq9.0000000000000337 PMC7870211

[3] da Silva PSL Reis ME Aguiar VE. Unplanned extubation in neonatal intensive care unit: A systematic review, critical appraisal and evidence-based recommendations. Respir Care. 2012;58:1237–1245. 23271815 10.4187/respcare.02164

[4] Carvalho FL Mezzacappa MA Calil R. Incidence and risk factors of accidental extubation in a neonatal intensive care unit. J Pediatr (Rio J). 2010;86:189–195. 20401427 10.2223/JPED.1999

[5] Hatch LD III Scott TA Slaughter JC. Outcomes, resource use, and financial costs of unplanned extubations in preterm infants. Pediatrics. 2020;145:e20192819. 32376726 10.1542/peds.2019-2819 PMC7263047

[6] Hatch LD Grubb PH Markham MH. Effect of anatomical and developmental factors on the risk of unplanned extubation in critically ill newborns. Am J Perinatol. 2017;34:1234–1240. 28494497 10.1055/s-0037-1603341 PMC5705226

[7] Hatch LD Scott TA Rivard M. Building the driver diagram: A mixed-methods approach to identify causes of unplanned extubations in a large neonatal ICU. Jt Comm J Qual Patient Saf. 2019;45:40–46. 30077484 10.1016/j.jcjq.2018.02.008

[8] Thomas RE Rao SC Minutillo C. Severe acquired subglottic stenosis in neonatal intensive care graduates: a case-control study. Arch Dis Child Fetal Neonatal Ed. 2018;103:F349–F354. 28866624 10.1136/archdischild-2017-312962

[9] Bertoni CB Bartman T Ryshen G. A quality improvement approach to reduce unplanned extubation in the NICU while avoiding sedation and restraints. Pediatr Qual Saf. 2020;5:e346. 34616962 10.1097/pq9.0000000000000346 PMC8487773

[10] Klugman D Melton K Maynord PO. Assessment of an unplanned extubation bundle to reduce unplanned extubations in critically ill neonates, infants, and children. JAMA Pediatr. 2020;174:e200268. 32282029 10.1001/jamapediatrics.2020.0268 PMC7154960

[11] Veldman A Trautschold T Weiss K. Characteristics and outcome of unplanned extubation in ventilated preterm and term newborns on a neonatal intensive care unit. Paediatr Anaesth. 2006;16:968–973. 16918660 10.1111/j.1460-9592.2006.01902.x

[12] Klugman D Berger JT Spaeder MC. Acute harm: unplanned extubations and cardiopulmonary resuscitation in children and neonates. Intensive Care Med. 2013;39:1333–1334. 23636827 10.1007/s00134-013-2932-x

[13] Aydon L Zimmer M Sharp M. Reporting the incidence of unplanned extubation in the neonatal intensive care unit. J Paediatr Child Health. 2018;54:784–787. 29476579 10.1111/jpc.13850

[14] Galiote JP Ridoré M Carman J. Reduction in unintended extubations in a level IV neonatal intensive care unit. Pediatrics. 2019;143:e20180897. 31028159 10.1542/peds.2018-0897

[15] Fontánez-Nieves TD Frost M Anday E. Prevention of unplanned extubations in neonates through process standardization. J Perinatol. 2016;36:469–473. 26796128 10.1038/jp.2015.219

[16] Loughead JL Brennan RA DeJuilio P. Reducing accidental extubation in neonates. Jt Comm J Qual Patient Saf. 2008;34:164125–164170. 10.1016/s1553-7250(08)34019-7 18419046

[17] Igo DA Kingsley KM Malaspina EM. Decreasing unplanned extubations in the neonatal ICU. Respir Care. 2021;66:1059–1062. 33975898 10.4187/respcare.08203

[18] Nelson MU Pinheiro JMB Afzal B. Experiences of a regional quality improvement collaborative to reduce unplanned extubations in the neonatal intensive care unit. Children (Basel). 2022;9:1180. 36010071 10.3390/children9081180 PMC9406401

[19] Holtrop JS Scherer LD Matlock DD. The importance of mental models in implementation science. Front Public Health. 2021;9:680316. 34295871 10.3389/fpubh.2021.680316 PMC8290163

[20] Ndakor SM Pezzano CJ Spilman L. Wide variation in unplanned extubation rates related to differences in operational definitions. J Patient Saf. 2022;18:e92–e96. 32398535 10.1097/PTS.0000000000000707

[21] Langley GJ MR Nolan KM. The Improvement Guide: A Practical Approach to Enhancing Organizational Performance. 2nd ed. Hoboken, N.J.: Jossey-Bass; 2009.

[22] American Society for Quality. Quality resources: Pareto chart. Available at https://asq.org/quality-resources/pareto-chart. Accessed October 19, 2023.

[23] Wong BM Sullivan GM. How to write up your quality improvement initiatives for publication. J Grad Med Educ. 2016;8:128–133. 27168874 10.4300/JGME-D-16-00086.1 PMC4857497

[24] Wiegmann DA Wood LJ Cohen TN. Understanding the “Swiss Cheese Model” and its application to patient safety. J Patient Saf. 2022;18:119–123. 33852542 10.1097/PTS.0000000000000810 PMC8514562

[25] Manges K Groves PS Farag A. A mixed methods study examining teamwork shared mental models of interprofessional teams during hospital discharge. BMJ Qual Saf. 2020;29:499–508. 10.1136/bmjqs-2019-009716 31776201

[26] Merkel L Beers K Lewis MM. Reducing unplanned extubations in the NICU. Pediatrics. 2014;133:e1367–e1372. 24777229 10.1542/peds.2013-3334

[27] Ferraz P Barros M Miyoshi M. Bundle to reduce unplanned extubation in a neonatal intensive care unit. J Matern Fetal Neonatal Med. 2020;33:3077–3085. 30632822 10.1080/14767058.2019.1568981

[28] Melton K Ryan C Saunders A. Reducing pediatric unplanned extubation across multiple ICUs using quality improvement. Pediatrics. 2022;149:e2021052259. 35490283 10.1542/peds.2021-052259

[29] Kandil SB Emerson BL Hooper M. Reducing unplanned extubations across a children’s hospital using quality improvement methods. Pediatr Qual Saf. 2018;3:e114. 31334446 10.1097/pq9.0000000000000114 PMC6581473

[30] P KL Nair V Vine M. Quality improvement study on new endotracheal tube securing device (Neobar) in neonates. Indian J Pediatr. 2017;84:20–24. 27641975 10.1007/s12098-016-2231-y

[31] Le Blanc G Jabbour E Patel S. Organizational risk factors and clinical impacts of unplanned extubation in the Neonatal Intensive Care Unit. J Pediatr. 2022;249:14–21.e5. 35714965 10.1016/j.jpeds.2022.06.012

[32] Nair V Smith H. Phased quality improvement interventions in reducing unplanned extubation in the neonatal ICU. Respir Care. 2020;65:1511–1518. 32291311 10.4187/respcare.07643

[33] Segura-Ramírez DK Fernández-Castiñeira S Gualotuña-Maigua DC. Neonatal unplanned extubations: an unsolved safety issue. Bol Med Hosp Infant Mex. 2021;78:259–264. 34107531 10.24875/BMHIM.20000255

