# Supplementary figures and images for: Synergizing Safety: A Customized Approach to Curtailing Unplanned Extubations through Shared Decision-making in the NICU
Source: Pediatr Qual Saf. 2024 May 9;9(3):e729. doi: 10.1097/pq9.0000000000000729 (PMC11093562; doi:10.1097/pq9.0000000000000729)

## COMPLIANCE WITH THE INTERVENTIONS

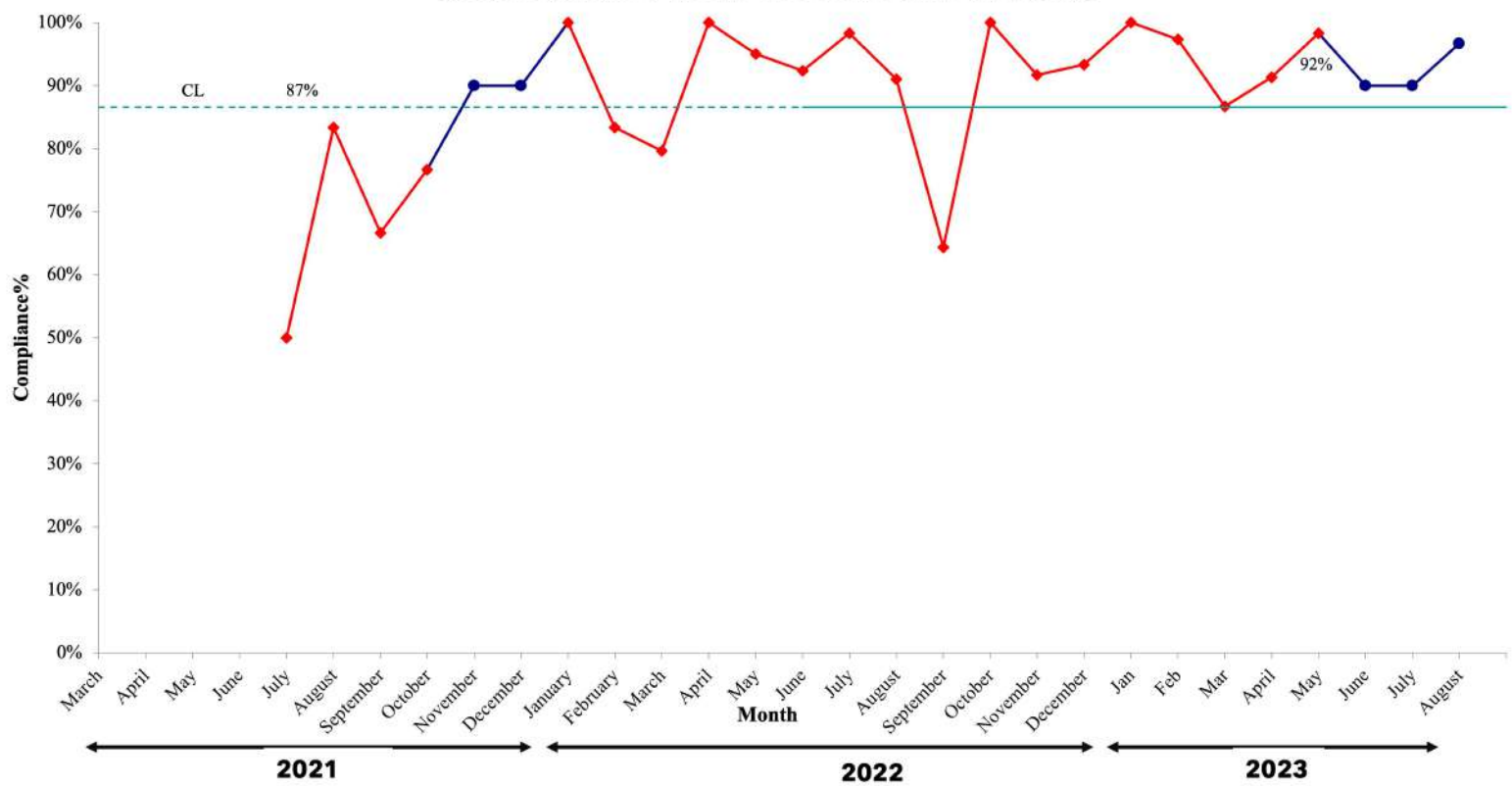

Supplement: Supplementary file 1 [file pqs-9-e729-s001.pdf]

Monthly UE events and number of ventilator days

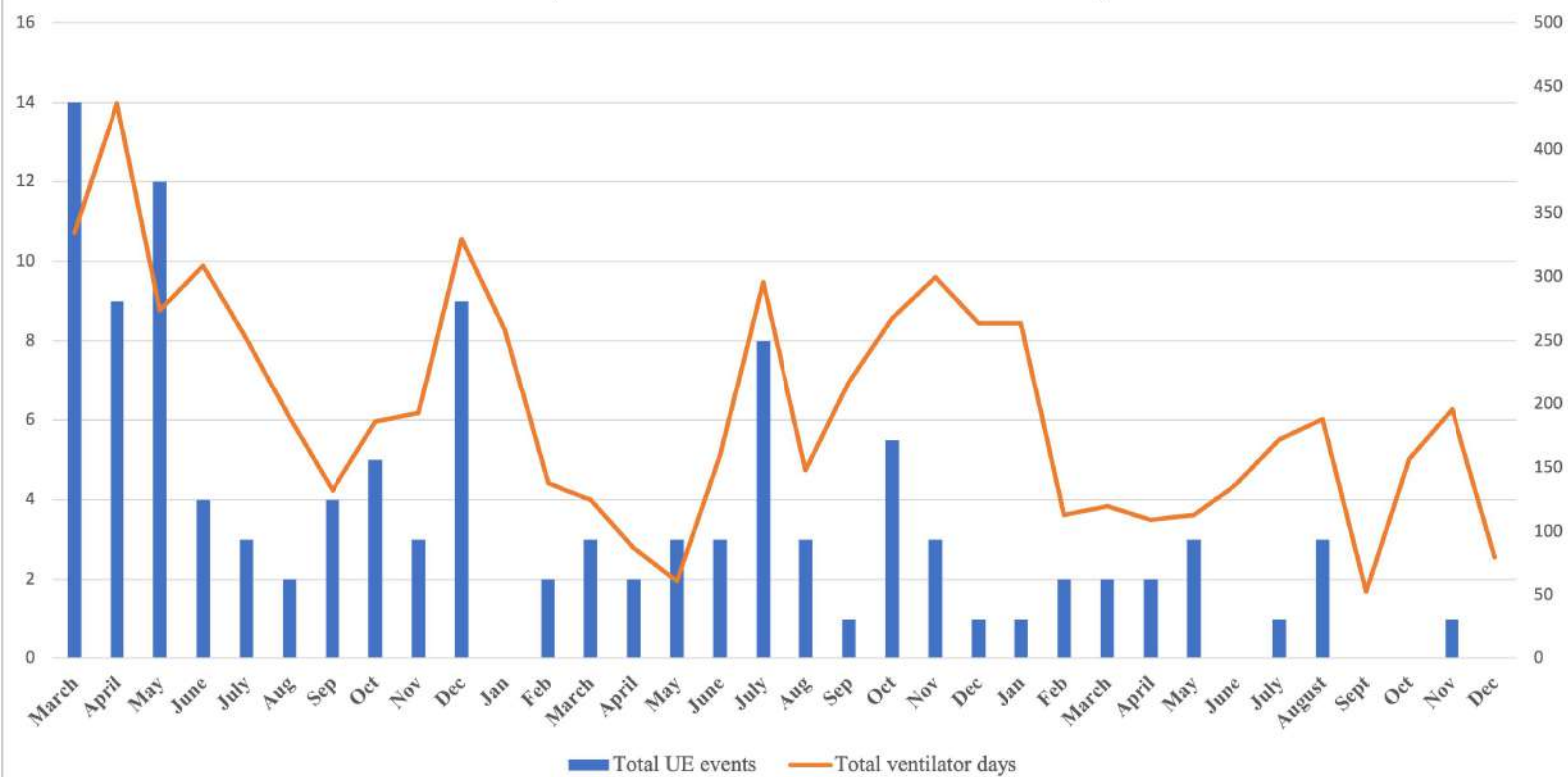

Supplement: Supplementary file 2 [file pqs-9-e729-s002.pdf]

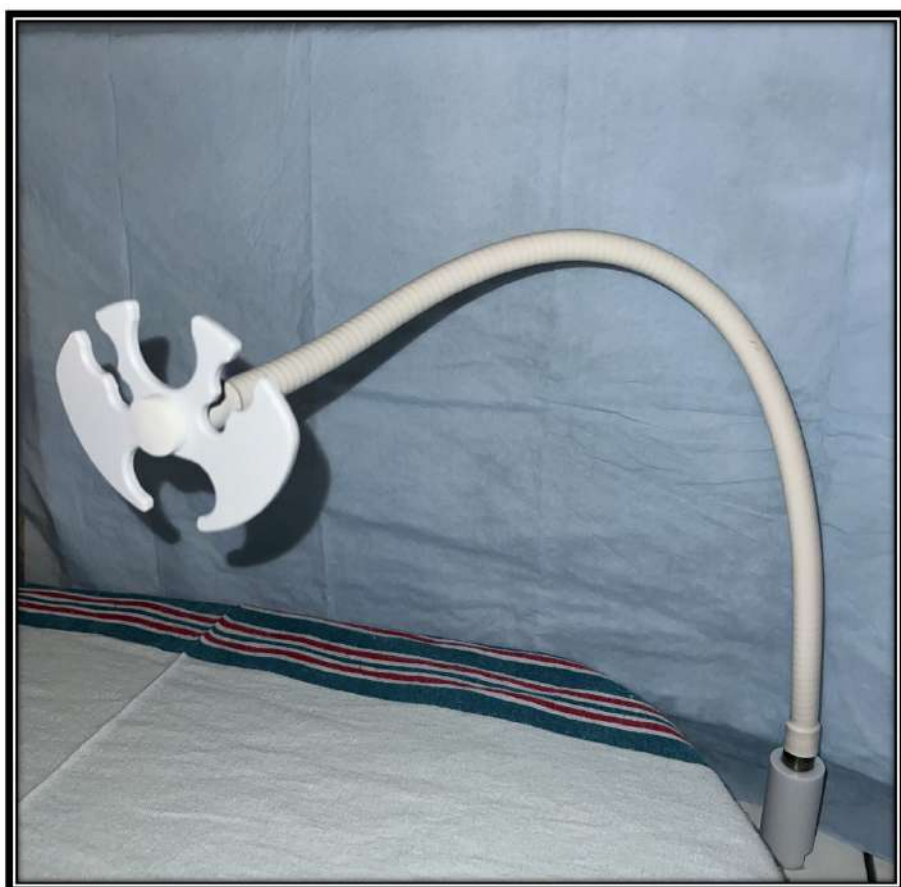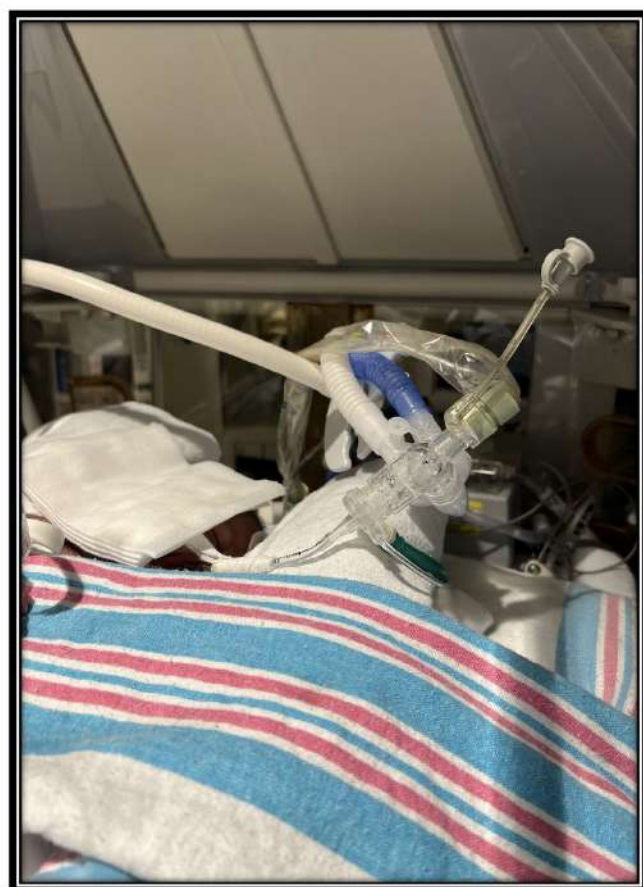

Supplement: Supplementary file 3 [file pqs-9-e729-s003.pdf]
